# Role of Lung Ultrasound in the Follow-Up of Children with Previous SARS-CoV-2 Infection: A Case-Control Assessment of Children with Long COVID or Fully Recovered

**DOI:** 10.3390/jcm12093342

**Published:** 2023-05-08

**Authors:** Danilo Buonsenso, Rosa Morello, Francesco Mariani, Cristina De Rose, Rossella Cortese, Luigi Vetrugno, Piero Valentini

**Affiliations:** 1 Department of Woman and Child Health and Public Health, Fondazione Policlinico Universitario A. Gemelli, IRCCS, 00168 Rome, Italy; 2 Global Health Center, Università Cattolica del Sacro Cuore, 00168 Rome, Italy; 3 School of Medicine, Università Cattolica del Sacro Cuore, 00168 Rome, Italy; 4 Department of Medical, Oral and Biotechnological Sciences, University of Chieti-Pescara, 66100 Chieti, Italy

**Keywords:** children, SARS-CoV-2 infection, Long COVID, lung ultrasound

## Abstract

Lung ultrasound (LUS) can detect lower respiratory tract involvement in children with acute SARS-CoV-2 infection. However, its role in follow-up assessments is still unclear. To describe LUS findings in children after SARS-CoV-2 infection, we conducted a prospective study in a population of pediatric patients referred to the post-COVID unit in a tertiary center during the study period from February 2021 to May 2022. Children were classified as recovered from acute infection or with persisting symptoms. LUS was performed in all children and a LUS score (ranging from 0 to 36 points) was calculated according to the Italian Academy of Thoracic Ultrasound. Six hundred forty-seven children (304 females, 47%) were enrolled. The median follow-up evaluation was two months. The median age was 7.9 (IQR: 6) years. At the follow-up evaluation, 251 patients (38.8%) had persistent symptoms, of whom 104 (16.1%) had at least one respiratory symptom. The median LUS level was 2 (IQR: 4). LUS findings and LUS scores did not differ in children with Long COVID compared to the group of children fully recovered from the initial infection. In conclusion, after SARS-CoV-2 infection, LUS was mostly normal or showed minimal artifacts in all groups of children.

## 1. Introduction

Since the first descriptions of SARS-CoV-2, our knowledge of the virus and the COVID-19 disease have gradually increased. Although the clinical impact of COVID-19 on adults and the elderly has been more relevant [1], children have not been spared from the pandemic, as they can develop a wide range of manifestations, from asymptomatic infection to severe disease and even mortality [2]. Additionally, children may be affected by post-acute sequelae, such as Multisystem Inflammatory Syndrome (MIS-C) and Long COVID [3]. In MIS-C, children develop a hyperacute systemic inflammatory response, while in Long COVID (LC), children report a non-specific cluster of more subtle symptoms including fever, cough, dyspnea, shortness of breath, gastrointestinal symptoms, fatigue, and muscle and articular pain [4]. In all these three spectra of disease, the lungs can be involved.

Pulmonary involvement in acute infection has been widely described in the literature [5], and lung ultrasound (LUS) has been proven able to detect lower respiratory tract infection (LRTI) during SARS-CoV-2 in both adults and children [6,7,8,9,10,11,12,13,14,15,16,17,18]. In the long-term follow-up after the initial infection, chronic lung involvement has been documented in adults, and LUS has been able to detect fibrotic lung changes or lung normalization of survivors [19,20]. In children, however, although they can also be affected by LC, the role of LUS in the follow-up after initial SARS-CoV-2 infection has not been rigorously evaluated yet. For these reasons, we performed this study to investigate the role of LUS in the follow-up of SARS-CoV-2 infection and to determine if children with Long COVID have a different lung involvement compared to those fully recovered.

## 2. Materials and Methods

### 2.1. Study Population

This was a prospective evaluation of children younger than 18 years of age with a previous microbiologically confirmed diagnosis (based on SARS-CoV-2 detected on a nasopharyngeal swab by real-time polymerase chain reaction, RT-PCR) of SARS-CoV-2 infection that were assessed in our pediatric post-COVID outpatient clinic in Rome, Italy. In our outpatient clinic, we evaluate children that had fully recovered from acute infection and those that presented with persisting symptoms. Children can be sent to the post-COVID unit after discharge from our institution, or they can be directly sent from family pediatricians (and therefore not seen at baseline during acute infection). We developed a protocol to assess children with LC, which has been described previously and includes a full LUS examination for all children [21]. The clinical assessments took place from 1 February 2021 to 31 May 2022.

Children with an initial microbiologically confirmed SARS-CoV-2 infection were referred to our pediatric post-COVID outpatient clinic for a clinical evaluation including full history, clinical, and LUS examination. In this context, children were diagnosed as “fully recovered” or with persisting symptoms (LC groups). The LC groups were further detailed, given the lack of an internationally agreed upon LC definition, as those having symptoms for at least 12 weeks (LC12 group) or those patients that specifically had persistent respiratory symptoms (LC respiratory group). This last group was included because LC is a disease with different clinical expressions; therefore, we wanted to be sure that those patients with persistent respiratory symptoms were properly characterized.

To summarize, the following categories of children were enrolled:

- Fully recovered children: This group included those that reported no persisting symptoms after acute SARS-CoV-2 infection at the time of follow-up post-onset of acute COVID-19 symptoms (at least 8 weeks);
- LC group: This group included any child with persisting symptoms after recovery from acute SARS-CoV-2 infection that could not be explained by an alternative diagnosis (excluded diagnoses—like anemia, autoimmunity, celiac disease, etc.—are detailed in our local protocol published elsewhere) [21]. Because of the lack of a case definition for LC when we started the study, we initially defined children with LC as those experiencing at least one persisting symptom for more than 2 weeks after the initial SARS-CoV-2 diagnosis;
- LC respiratory group: This group included any child with persisting respiratory symptoms for at least 2 weeks after SARS-CoV-2 infection that could not be explained by an alternative diagnosis;
- LC12 group: During the study period, the WHO released a definition of LC in adults, which defined LC as symptoms persisting for 12 weeks or more [22], and a recent Delphi proposed a similar definition for children [23]. Therefore, we implemented a further subgroup fulfilling this definition.

Disease severity during acute infection was classified as asymptomatic, mild, moderate, or severe, according to the adapted classification by Buonsenso et al. [24].

### 2.2. Inclusion and Exclusion Criteria

The following inclusion criteria were used:

- Children aged 0–18 years;
- The child presented in a primary or secondary care medical facility due to COVID-19 illness;
- Laboratory (RT-PCR) diagnosis of acute SARS-CoV-2 infection;
- Assessed at least 2 weeks after the first positive test for SARS-CoV-2 PCR;
- Patients referred to our post-COVID unit during the study period;
- Parent’s/carer’s/guardian’s consent to participate.

Exclusion criteria:

- Children with chronic respiratory conditions before the initial SARS-CoV-2 infection (e.g., uncontrolled asthma, frequent wheezing, cystic fibrosis, and other chronic primary lung diseases that may negatively impact LUS pattern independently from SARS-CoV-2 infection);
- Children with confirmed or suspected primary or acquired immune compromising conditions, the recent or current administration of immune suppressive therapies, or other diseases affecting the immune system, which may indirectly negatively impact LUS pattern independently from SARS-CoV-2 infection, due to previous or recent respiratory infections;
- Patients that in the time between initial SARS-CoV-2 infection and LUS examination had at least one documented viral/bacterial lower respiratory tract infection. Such individuals were excluded since this new infection may negatively impact LUS pattern independently from SARS-CoV-2 infection;
- Patients evaluated outside the study period;
- Children without parent’s/carer’s/guardian’s consent to participate.

### 2.3. Outcomes

The primary aim of the study was to describe the LUS findings of children evaluated at least two weeks after the initial infection. Given that these data were unknown at the time of the study design, this was set as a pilot study, and no sample size calculation was implemented.

Secondary aims were to evaluate LUS findings in children fully recovered from the infection or with LC; to evaluate LUS findings in children fully recovered from the infection or with persisting respiratory symptoms (LC respiratory group); and to evaluate LUS findings in children fully recovered from the infection or with persisting symptoms for at least 12 weeks (LC12).

### 2.4. Ultrasound Examination

Three pediatricians working together in clinical and research LUS practice for more than 5 years, making teaching modules together and sharing the same methodology for 5 years, performed the LUS examinations. LUS was performed with Esaote MyLab Eight using a 10-12 MHz linear probe in children in a sitting position on the examination bed.

Our pediatric LUS COVID team used, for the assessment of patients with COVID-19 (during both acute infection and follow-up), a standardized approach concerning equipment and acquisition protocol, as previously described by Soldati et al. [25]. Soldati et al.’s approach requires the division of the patient’s chest into 14 areas (3 posterior, 2 lateral, and 2 anterior areas on each side) and a single intercostal scan of each area. These 14 areas (3 posterior, 2 lateral, and 2 anterior for each lung) were scanned for each patient, registering one picture for each area.

**Figure 1:**
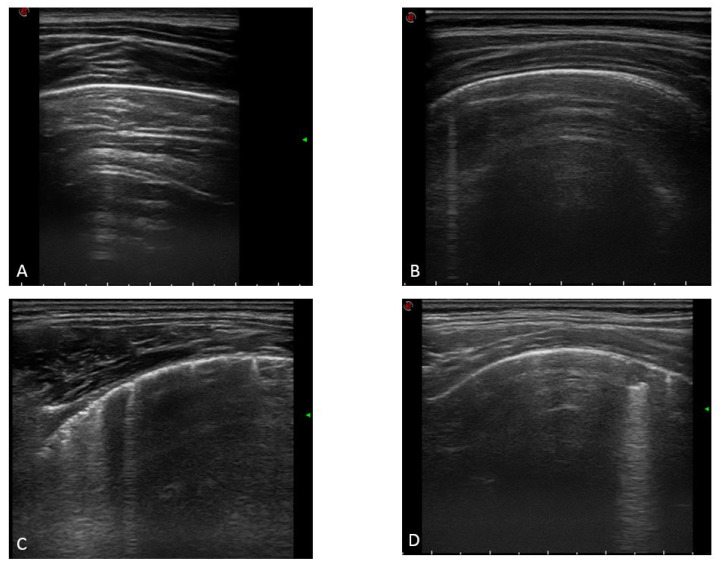
(**A**) Pleural A lines in a normal LUS examination; (**B**) regular pleural line with a single vertical artifact; (**C**) irregular pleural line with multiple confluent vertical artifacts; and (**D**) small (less than 1 cm deep) subpleural consolidation.

Then, each area was assigned a numeric score from 0 to 3 depending on the severity of findings (as shown in Figure 1): 0 = normal LUS examination; 1 = the pleural line is a regular or irregular pleural line with visible non-confluent vertical artifacts; 2 = irregular pleural line with multiple confluent vertical artifacts and/or small (less than 1 cm deep) subpleural consolidations; and 3 = dense and largely extended areas of white lung with or without larger consolidations (1 cm deep or more). Such a score gives a minimum of 0 (no lung involvement, LUS normal) and a maximum of 36 (worse lung involvement).

Moreover, we collected how many patients had one or more areas with a score of 2 and did the same for those with a score of 3. In addition, we calculated the mean scores of all study populations for each lung area and a total score for all 14 areas. For each area, the author performing the exam, with subsequent confirmation by at least two other authors reviewing the recorded clip, assigned the score. Disagreement was resolved through discussion among all authors.

### 2.5. Statistical Analysis

For continuous variables, the Kolmogorov–Smirnov test was used to assess whether the distribution was normal or not. Categorical variables were reported as count and percentage. Continuous variables with normal distribution were expressed as means with standard deviations; data with a skewed distribution were expressed as medians with an interquartile range (IQR 75–25%). The statistical association between categorical variables was obtained by Chi-squared tests or Fisher’s exact tests. The Mann–Whitney U-test was used to assess differences in two groups for continuous variables if not normally distributed; a post hoc analysis was performed to adjust *p*-values for multiple comparisons with the Benjamini–Hochberg test. A *p*-value < 0.05 was considered statistically significant.

## 3. Results

### 3.1. Study Population and Clinical Characteristics

During the study period, 647 children (304 females, 47%) were enrolled. The median age of the children was 7.9 (IQR: 6) years; 96 of them (14.8%) were affected by other comorbidities, and 537 patients were not vaccinated for SARS-CoV-2 before the COVID infection. Regarding the SARS-CoV-2 infection, 32 children (4.9%) were asymptomatic, 600 patients (92.8%) had a mild form, 13 patients (2%) had a moderate form, and 2 (0.3%) had a severe form. Details about demographics and clinical characteristics of acute infection are reported in Table 1.

At the follow-up evaluation, 251 patients (38.8%) presented with a persistence of symptoms, and 104 patients (16.1%) had the persistence of at least one respiratory symptom. In particular, 9 children (1.4%) presented fever, 21 children (3.2%) had rhinitis/nasal congestion, 8 children (1.2%) had anosmia, 7 children (1.1%) had dysgeusia, 25 children (3.9%) had a cough, 4 children (0.6%) had dyspnea at rest, 55 (8.5%) had dyspnea after exercise, 8 children (1.2%) had new-onset asthma, 24 children (3.7%) had chest pain, 17 children (2.6%) had tachycardia, 26 children (4%) had articular pain, 44 children (6.8%) had muscle pain, 57 children (8.8%) had a headache, 40 children (6.2%) had diarrhea and other gastrointestinal symptoms, 11 (1.7%) had a rash, and 127 children (19.6%) had other symptoms (Table 2).

### 3.2. LUS Findings

All patients performed LUS. The median LUS level was 2 (IQR: 4). The most affected pulmonary zones were the lateral zones (23.5%). In comparison, the posterior zones were affected in 119 patients (18,4%), the anterior zones were affected in 142 patients (21.9%), lateral and anterior zones were affected in 33 patients (5.1%), posterior and anterior zones were affected in 3 patients (0.5%), posterior and lateral zones were affected in 3 patients (0.5%), all three zones were affected in 2 patients (0.3%), and 195 patients (30.1%) had no zone affected. The median of the mean LUS level of the posterior zone was 0 (IQR: 0.17), the median of the lateral zone was 0 (IQR: 0.25), and the medians of the anterior zones were 0 (IQR: 0.25). Between all patients, 207 children (32%) presented at least one pulmonary zone with an LUS score of 2, 66 patients (10.2%) had at least two zones with an LUS score of 2, 1 patient (0.2%) had at least one pulmonary zone with an LUS score of 3, and no patients had at least two pulmonary zones with an LUS score of 3.

A comparison between the LUS characteristics of patients with a persistence of respiratory symptoms and patients without a persistence of respiratory symptoms is reported in Table 3. From the Mann–Whitney analysis, the only statistically significant difference in the ultrasound parameters between those two groups was observed for the average LUS level of the lateral zones (*p* = 0.03). A statistically significant difference in the age of patients was also observed in those two groups (10.29 (IQR: 7.83) vs. 7.58 (IQR: 5.75) *p* < 0.0001). The post hoc analysis performed to correct the *p*-value for multiple analyses found no statistically significant differences in LUS findings in the two groups.

Table 4 reports the comparison of the LUS characteristics between patients that complained of any persisting symptoms at any follow-up and those who fully recovered. Table 5 shows a comparison of LUS characteristics between patients with persisting symptoms at a follow-up of at least 12 weeks (LC12 group) and children that fully recovered. In both scenarios, LUS was mostly normal in all patients, and no statistically significant differences were found in children with either persisting symptoms or fully recovered.

## 4. Discussion

In this study, we assessed LUS findings in children evaluated after the initial SARS-CoV-2 infection. Overall, we found that almost all children had either a normal LUS pattern or minimal artifacts, suggesting complete lung recovery after the initial infection. Importantly, LUS findings were similar in both recovered children and those that developed Long COVID.

As COVID-19 pneumonia mainly affects the peripheral areas of the lungs [5], studies in both adults and children have demonstrated that LUS could detect low respiratory tract infection (LRTI) during SARS-CoV-2 [6,7,8,9,10,11,12,13,14,15,16,17,18]. During pediatric acute infection, LUS can document vertical artifacts or, in more severe cases, subpleural consolidations. For these reasons, there was a rational for its use even in the follow-up of patients with COVID-19, and in fact, there is extensive experience in adult practice. Long COVID is defined as the persistence of symptoms after 4 weeks of acute infection, while “post-COVID syndrome” is defined as the persistence of symptoms after 12 weeks of acute infection in adults [26]. Both these syndromes offer another opportunity to spread LUS use even more. With ultrasound imaging, adults with these syndromes can be examined with a tremendous advantage, as the first-line follow-up is available at lower costs and without radiation exposure. Sonnweber et al. showed that in 145 patients, 41% of all subjects exhibited persistent symptoms 100 days after COVID-19 onset. Dyspnea was the most frequent (36%), and cardiac impairment, including reduced left ventricular function or signs of pulmonary hypertension, was only present in a minority of cases [27]. Guler et al. in a study that included 113 critically ill COVID-19 survivors, confirmed that several comorbidities were associated with impaired pulmonary function due to small-airway and lung parenchymal disease [28]. B-lines associated with pleural line disruption and thickness are the characteristic element and positively correlate with HRCT score, FVC, and DLCO in idiopathic fibrosis [29]. LUS showed significant superiority over chest X-rays and, most importantly, showed similar sensitivity and negative predictive value compared to CT [30]. Consequently, a normal LUS examination could rule out the presence of lung involvement in adults. An experienced sonographer can do this examination within 5 min and brief training [29]. However, we need to remember that B-lines’ sensitivity is high, but their specificity may differ. Therefore, appropriate training is needed to properly interpret artifacts.

Given the large number of adults with COVID-19 followed up with LUS, we implemented our protocol based on the rational that LUS may help recognize recovery or persisting peripheral lung pathology. The rational was also based on a small study we previously published on COVID-19 children [11] and also on our local practice (we use LUS routinely to follow up patients with pneumonia and bronchiolitis) [31]. In contrast to adult data, which are characterized by a more severe lung involvement during acute infection, more reassuring data are available for children, although fewer studies have assessed this issue. La Regina et al. evaluated 607 children and showed that about 8% of children had subpleural consolidations. However, the authors did not provide sufficient LUS score data to understand overall lung involvement [32]. In addition, they did not exclude children with chronic respiratory conditions such as asthma, which themselves can be associated with LUS abnormalities [9]. In this regard, our data, with a probably better-selected cohort, are more reassuring and show that children present mostly normal LUS findings, with mean scores of 2. Our data are in line with the observations that the large majority of children develop a mild disease during acute infection and, consequently, chronic lung involvement remains minimal at follow-up.

An important finding of our study is that LUS was mostly normal or only found minimal pathological findings of unclear significance in all patients, with no differences when we compared children with Long COVID or who recovered, even when we created a subgroup of Long COVID children with only respiratory symptoms. These findings, however, are not unexpected in children for two reasons. First, acute COVID-19 is known to be a mild disease in the large majority of children with severe/critical disease being extremely rare [4]. As such, significant lung involvement during acute infection is rare and, therefore, it is not unexpected that during follow-up, LUS is mostly normal. Regarding those with Long COVID, there is increasing evidence that this subgroup of patients shows similarities with other post-viral chronic fatigue syndromes whose pathogenesis is not yet fully elucidated, although there is increasing evidence that functional mechanisms, rather than organ damage, may play a role [33]. For example, recent advances suggest that cellular events leading to chronic stimulation of immune responses, viral persistence, gut dysbiosis, endothelial inflammation of peripheral microcirculation and microclots, and mitochondrial dysfunction may play a role in the pathogenesis of Long COVID [33]. Therefore, more advanced imaging modalities, such as technologies that provide information about organ functioning, could play a more significant role in characterization of pediatric Long COVID [34,35]. In this regard, LUS is, in our opinion, mostly an instrument that may allow a radiation-free assessment of the peripheral lung and help the clinician determine full recovery after initial infection using an easy point-of-care technology.

This study has limitations to address. First, we did not perform LUS during acute infection in these children. Therefore, we cannot conclude that any pathological LUS feature found at follow-up is strictly due to the previous SARS-CoV-2 infection, since we cannot exclude that some features were pre-existent or due to other intercurrent infection (although we excluded patients that had new infections between COVID-19 and our follow-up visit). Additionally, follow-up times differed in our cohort, and for this reason, we created several subgroups, including a cohort of children with a stricter definition of Long COVID. Lastly, we did not include a control group of otherwise healthy children that never had COVID-19, and we therefore cannot conclude if the minimal pathological LUS findings we documented can be para-physiologic and also present in healthy children or whether they are specific outcomes of the previous infection.

## 5. Conclusions

In conclusion, in this large cohort of children followed up after SARS-CoV-2 infection, LUS was mostly normal in all children or only showed minimal artifacts, suggesting that children recover from infection without presenting clinically relevant LUS findings as may happen in adults. Importantly, LUS findings were similar in children that either recovered or developed persisting symptoms, suggesting that LUS is not a useful tool for the characterization of children with Long COVID.

## Figures and Tables

**Table 1:** Demographics and clinical characteristics of acute COVID infection in the study population.

	Study Population (N = 647)
Gender	
Male	343 (53%)
Female	304 (47%)
Comorbidity	
No	551 (85.2%)
Yes	96 (14.8%)
Severity	
Asymptomatic	32 (4.9%)
Mild	600 (92.8%)
Moderate	13 (2%)
Severe	2 (0.3%)
Hospitalization	
No	634 (98%)
Yes	13 (2%)
Intensive Care	
No	645 (99.7%)
Yes	2 (0.3%)
Fever	
No	202 (31.2%)
Yes	445 (68.8%)
Rhinitis/Nasal Congestion	
No	378 (58.4%)
Yes	269 (41.6%)
Anosmia	
No	601 (92.9%)
Yes	46 (7.1%)
Dysgeusia	
No	610 (94.3%)
Yes	37 (5.7%)
Cough	
No	439 (67.9%)
Yes	208 (32.1%)
Dyspnea at Rest	
No	636 (98.3%)
Yes	11 (1.7%)
Dyspnea After Exercise	
No	633 (97.8%)
Yes	14 (2.2%)
Asthma	
No	643 (99.4%)
Yes	4 (0.6%)
Chest Pain	
No	628 (97.1%)
Yes	19 (2.9%)
Articular Pain	
No	595 (92%)
Yes	52(8%)
Muscle Pain	
No	578 (89.3%)
Yes	69 (10.7%)
Asthenia	
No	466 (72%)
Yes	181 (28%)
Headache	
No	462 (71.4%)
Yes	185 (28.6%)
Diarrhea and/or Other Gastrointestinal Symptoms	
No	541 (83.6%)
Yes	106 (16.4%)
Rash	
No	626 (96.8%)
Yes	21 (3.2%)

**Table 2:** Clinical characteristics in the follow-up of patients with previous COVID infection in the study population.

	Study Population (N = 647)
Hospitalization	
No	634 (98%)
Yes	13 (2%)
Intensive Care	
No	645 (99.7%)
Yes	2 (0.3%)
Persistent Symptoms at Follow-Up	
No	396 (61.2%)
Yes	251 (38.8%)
Chronic Unexplained Fever	
No	638 (98.6%)
Yes	9 (1.4%)
Rhinitis/Nasal Congestion	
No	626 (96.8%)
Yes	21 (3.2%)
Anosmia	
No	639 (98.8%)
Yes	8 (1.2%)
Dysgeusia	
No	640 (98.9%)
Yes	7 (1.1%)
Cough	
No	622 (96.1%)
Yes	25 (3.9%)
Dyspnea at Rest	
No	643 (99.4%)
Yes	4 (0.6%)
Dyspnea After Exercise	
No	592 (91.5%)
Yes	55 (8.5%)
Asthma	
No	639 (98.8%)
Yes	8 (1.2%)
Chest Pain	
No	623 (96.3%)
Yes	24 (3.7%)
Tachycardia	
No	630 (97.4%)
Yes	17 (2.6%)
Articular Pain	
No	621 (96%)
Yes	26 (4%)
Muscle Pain	
No	603 (93.2%)
Yes	44 (6.8%)
Headache	
No	590 (91.2%)
Yes	57 (8.8%)
Diarrhea and/or Other Gastrointestinal Symptoms	
No	607 (93.8%)
Yes	40 (6.2%)
Rash	
No	636 (98.3%)
Yes	11 (1.7%)
Other Symptoms	
No	520 (80.4%)
Yes	127 (19.6%)

**Table 3:** Comparison of ultrasound characteristics between patients with a persistence of respiratory symptoms and patients without a persistence of respiratory symptoms.

	Persistence of Respiratory Symptoms (N = 104)	Absence of Respiratory Symptoms (N = 543)	*p*-Value	Adjusted *p*-Value
Age (years) (median, IQR)	10.29 (7.83)	7.58 (5.75)	0.0001	0.0005
LUS score (median, IQR)	2 (4)	2 (3)	0.33	0.55
Average LUS value of the posterior zones (median, IQR)	0 (0.17)	0 (0.17)	0.74	0.93
Average LUS value of the lateral zones (median, IQR)	0 (0.5)	0 (0.25)	0.03	0.08
Average LUS value of the anterior zones (median, IQR)	0 (0.25)	0 (0.25)	0.95	0.95
Presence of at least one zone with an LUS level of 2 (*n*, %)				
Yes	37 (35.6%)	170 (31.3%)		
No	67 (64.4%)	373 (68.7%)	0.39	
Presence of at least two zones with an LUS level of 2 (*n*, %)				
Yes	16 (15.4%)	50 (9.2%)		
No	88 (84.6%)	493 (90.8%)	0.05	
Presence of at least one zone with an LUS level of 3 (*n*, %)				
Yes	0 (0%)	1 (0.2%)		
No	104 (100%)	542 (99.8%)	1	

**Table 4:** Comparison of ultrasound characteristics between patients with a persistence of symptoms and patients without a persistence of symptoms at follow-up evaluation.

	Persistence of Symptoms (N = 251)	Absence of Symptoms (N = 396)	*p*-Value
Presence of at least one zone with an LUS level of 2 (*n*, %)			
Yes	88 (35.1%)	119 (30.1%)	
No	163 (64.9%)	277 (69.9%)	0.18
Presence of at least two zones with an LUS level of 2 (*n*, %)			
Yes	30 (12%)	36 (9.1%)	
No	221 (88%)	360 (90.9%)	0.24
Presence of at least one zone with an LUS level of 3 (*n*, %)			
Yes	0 (0%)	1 (0.3%)	
No	251 (100%)	395 (99.7%)	1

**Table 5:** Comparison of echography characteristics between patients that fulfilled the Long COVID definition (LC12 group) and patients who did not.

	LC12 Group (N = 99)	Non-LC12 Group (N = 548)	*p*-Value
Presence of at least one zone with an LUS level of 2 (*n*, %)			
Yes	65 (65.7%)	173 (31.6%)	
No	34 (34.3%)	375 (68.4%)	0.59
Presence of at least two zones with an LUS level of 2 (*n*, %)			
Yes	11 (11.1%)	55 (10%)	
No	88 (88.9%)	493 (90%)	0.74
Presence of at least one zone with an LUS level of 3 (*n*, %)			
Yes	0 (0%)	1 (0.2%)	
No	99 (100%)	547 (99.8%)	1

## Data Availability

The data presented in this study are not publicly available but are available on request from the corresponding author.
